# Inhaled “Muco‐Trapping” Monoclonal Antibody Effectively Treats Established Respiratory Syncytial Virus (RSV) Infections

**DOI:** 10.1002/advs.202306729

**Published:** 2024-01-15

**Authors:** Morgan D. McSweeney, Sarhad Alnajjar, Alison M. Schaefer, Zach Richardson, Whitney Wolf, Ian Stewart, Pun Sriboonyapirat, Justin McCallen, Ellen Farmer, Bernadette Nzati, Sam Lord, Brian Farrer, Thomas R. Moench, Priya A. Kumar, Harendra Arora, Raymond J. Pickles, Anthony J. Hickey, Mark Ackermann, Samuel K. Lai

**Affiliations:** ^1^ Inhalon Biopharma Research Triangle Park NC 27707 USA; ^2^ Department of Comparative Biomedical Sciences, School of Veterinary Medicine, Faculty of Health and Medical Sciences University of Surrey Guildford GU2 7AL UK; ^3^ Division of Pharmacoengineering and Molecular Pharmaceutics, Eshelman School of Pharmacy University of North Carolina‐Chapel Hill Chapel Hill NC 27599 USA; ^4^ RTI International Research Triangle Park NC 27709 USA; ^5^ Oregon State University Corvallis Oregon 97331 USA; ^6^ Department of Anesthesiology, School of Medicine University of North Carolina Chapel Hill NC 27599 USA; ^7^ Outcomes Research Consortium Cleveland OH 44195 USA; ^8^ Department of Anesthesiology University of Mississippi Medical Center Jackson MS 39216 USA; ^9^ Department of Microbiology & Immunology, School of Medicine University of North Carolina at Chapel Hill Chapel Hill NC 27599 USA; ^10^ USDA/ARS‐National Animal Disease Center Ames IA 50010 USA

**Keywords:** mAb nebulization, monoclonal antibody, nebulization, respiratory syncytial virus, RSV

## Abstract

Respiratory syncytial virus (RSV) causes substantial morbidity and mortality in infants, the immunocompromised, and the elderly. RSV infects the airway epithelium via the apical membrane and almost exclusively sheds progeny virions back into the airway mucus (AM), making RSV difficult to target by systemically administered therapies. An inhalable “muco‐trapping” variant of motavizumab (Mota‐MT), a potent neutralizing mAb against RSV F is engineered. Mota‐MT traps RSV in AM via polyvalent Fc‐mucin bonds, reducing the fraction of fast‐moving RSV particles in both fresh pediatric and adult AM by ≈20–30‐fold in a Fc‐glycan dependent manner, and facilitates clearance from the airways of mice within minutes. Intranasal dosing of Mota‐MT eliminated viral load in cotton rats within 2 days. Daily nebulized delivery of Mota‐MT to RSV‐infected neonatal lambs, beginning 3 days after infection when viral load is at its maximum, led to a 10 000‐fold and 100 000‐fold reduction in viral load in bronchoalveolar lavage and lung tissues relative to placebo control, respectively. Mota‐MT‐treated lambs exhibited reduced bronchiolitis, neutrophil infiltration, and airway remodeling than lambs receiving placebo or intramuscular palivizumab. The findings underscore inhaled delivery of muco‐trapping mAbs as a promising strategy for the treatment of RSV and other acute respiratory infections.

## Graphical abstract

Respiratory syncytial virus (RSV) is the most common cause of hospitalizations in children under the age of 1. To date, there is no effective treatment for established RSV infections, only methods for prevention. In this research, the authors developed an inhaled anti‐RSV treatment that showed efficacy in mice, rats, and a neonatal lamb model of RSV infection, significantly reducing viral load and pathology compared to placebo‐treated animals.

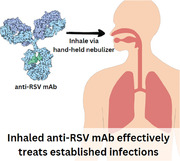

## 1 Introduction

Respiratory syncytial virus (RSV) is the most common cause of lower respiratory tract infections (LRTIs) in young children, and a major cause of morbidity and mortality in both the immunocompromised and the elderly.^[^ ^1^, ^2^ ^]^ Globally, RSV causes ≈35–40 million cases of acute LRTIs in children under the age of 5 each year.^[^ ^1^ ^]^ In the U.S., there are ≈2 million children under 5 that have RSV infections requiring medical attention each year, with ≈2–3% of those (i.e., ≈40 000–60 000) culminating in hospitalization.^[^ ^3^ ^]^ While deaths from RSV are relatively rare in high‐income countries, RSV is responsible for substantial infant morbidity and health care utilization, including ≈20% of all hospitalizations and ≈18% of all emergency department visits by infants from November through April each year.^[^ ^3^ ^]^ Likewise, RSV causes major morbidity^[^ ^4^, ^5^, ^6^ ^]^ and mortality^[^ ^7^, ^8^, ^9^ ^]^ in adults who have compromised immune systems and in older adults.

While a number of vaccines are in late‐stage clinical development and some have been approved, their long‐term efficacy and safety remain to be determined. The COVID experience also highlights the challenge with vaccine hesitancy and underscores the need for safe and effective treatments, both for breakthrough infections and for those who choose to not receive vaccination or prophylaxis. Not surprisingly, numerous RSV treatments have been advanced into clinical development, encompassing both small molecule antivirals and biologics. None have succeeded to date. Many small molecule antivirals have failed due to either safety concerns or viral escape.^[^ ^10^, ^11^ ^]^ Although palivizumab (Synagis; a monoclonal antibody (mAb) against RSV F protein) and now nirsevimab are safe and afford modest‐to‐good efficacy (≈50‐70%) when used as a prophylactic,^[^ ^12^, ^13^ ^]^ systemically dosed mAbs have not been effective as a treatment for RSV infection. These realities motivated us to explore alternative strategies in advancing a safe and effective treatment for RSV.

Interestingly, virtually all prior failed investigational therapies were administered by systemic or oral dosing of the treatment. Such delivery does not address the unique pathophysiology of RSV, where infections are primarily confined to the airways: the respiratory epithelium predominantly sheds progeny RSV virions into the airway mucus (AM) overlaying the epithelium.^[^ ^14^, ^15^, ^16^ ^]^ There is no appreciable direct cell‐to‐cell transmission or basolateral spread of RSV. This unique pathophysiology creates an opportunity to harness the natural clearance of AM to quickly remove RSV from the airways. AM is continuously secreted along the airways, cleared by ciliary beating or coughing, is then swallowed, and finally sterilized by gastric acid.^[^ ^17^, ^18^ ^]^ This motivated us to engineer mAbs against RSV with a “muco‐trapping” effector function, whereby antigen‐specific IgGs with elevated levels of G0/G0F N‐glycans can trap pathogens in the AM through a network of many Fc‐mucin crosslinks.^[^ ^19^ ^]^ We have previously employed the same strategy to engineer mAbs that can effectively trap various pathogens in mucus,^[^ ^20^, ^21^, ^22^, ^23^, ^24^, ^25^ ^]^ including similar respiratory viruses such as SARS‐CoV‐2.^[^ ^26^ ^]^ Here, we report motavizumab, engineered with “muco‐trapping” Fc, affords effective inhaled therapy against established RSV infections in both small and large animal models.

## 2 Results

### 2.1 Inhaled mAb Effectively Immobilizes RSV in Human AM in Fc‐N‐Glycan Dependent Manner

To explore whether Fc‐mucin interactions could be harnessed to immobilize RSV in AM, we elected to investigate motavizumab, an affinity‐matured variant of the FDA‐approved palivizumab that targets a well‐conserved site II epitope on RSV F‐protein,^[^ ^27^, ^28^ ^]^ and which has been safely dosed into thousands of infants in prior clinical trials.^[^ ^27^, ^29^, ^30^, ^31^, ^32^, ^33^ ^]^ We produced motavizumab with Fc‐N‐glycosylation yielding elevated levels of G0F (i.e., terminating in N‐acetylglucosamine on each branch rather than terminal galactose or sialic acid), which we previously found to facilitate potent mAb‐mediated trapping in mucus^[^ ^24^ ^]^; we term this molecule “Mota‐MT”. To measure its muco‐trapping potency, we fluorescently labeled purified RSV‐A2 virions and employed multiple particle tracking (MPT) to quantify the motion of hundreds to thousands of individual virions in each aliquot of fresh human AM collected from extubated endotracheal tubes from adult and pediatric patients receiving non‐pulmonary surgery. Fluorescent labeling of the virions did not interfere with mAb binding, reducing the total mAb that bound RSV by only ≈10%−20%, per ELISA analysis.^[^ ^19^ ^]^

**Figure 1:**
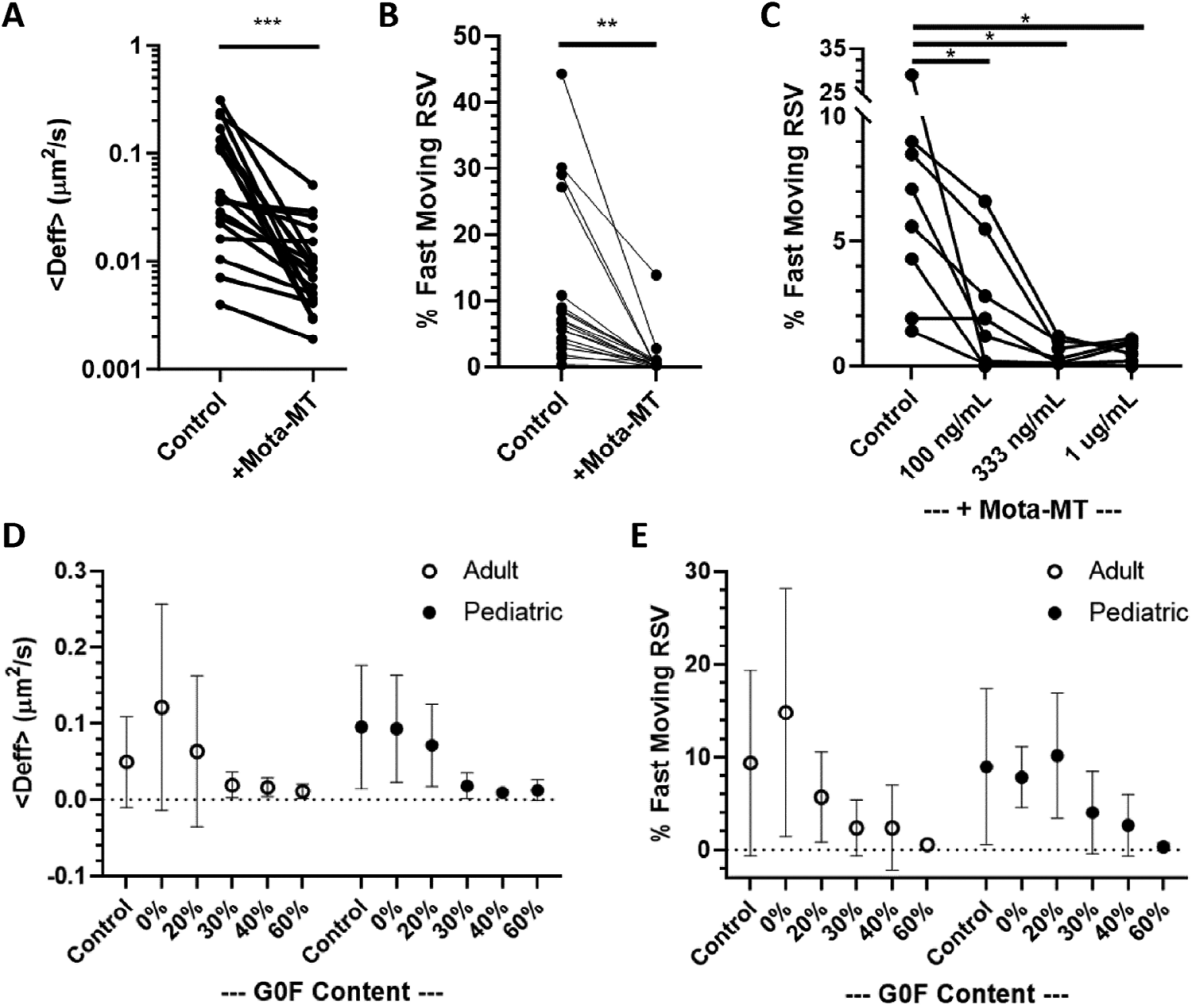
mAb‐mediated trapping of RSV in human adult and pediatric AM. Fresh AM were collected from extubated endotracheal tubes. The motion of hundreds of individual fluorescently‐tagged RSV virions per condition were captured by video microscopy at 15 Hz and sub‐100 nm spatiotemporal resolution, and quantified by multiple particle tracking. Mota‐MT markedly reduces the A) effective diffusivities <Deff> and B) fraction of fast‐moving RSV virions in human AM (*n* = 11 adult AM samples and *n* = 8 pediatric AM samples). C) Percent of virions that were fast moving, in different concentrations of Mota‐MT added to adult (*n* = 5) and pediatric (*n* = 3) AM. The reduced mobility is dependent on the dose of of Mota‐MT added to the AM. D,E) Increased Fc‐G0F content enhances the effectiveness of Mota‐mediated trapping of RSV in both adult (*n* = 6) and pediatric (*n* = 7) human AM. Statistical comparisons are not graphed in panels D and E, and are instead described in the Results text for clarity. *, **, and *** represent *p*<0.05, 0.01, and 0.001, respectively.

In freshly collected native adult and pediatric AM samples, we observed a substantial fraction of RSV virions exhibiting substantial mobility (Movie S1, Supporting Information). There were substantial variations both within the same AM specimen and across AM specimens from different donors; this is reflected by the range of the ensembled‐averaged effective diffusivities (<Deff>) measured via MPT; across all AM samples, the average <Deff> was 0.074 µm^2^ s^−1^ (SD, 0.073) (**Figure** **1**A). Given this heterogeneity, we further quantified the fraction of virions possessing sufficient mobility to diffuse through a 50 µm thick mucus layer within 30 mins, which we classified as “fast‐moving” (Figure 1B). We found on average there were ≈9.1% (SD, 8.8%) of RSV virions classified as “fast‐moving” across different AM specimens. These results are in agreement with the capacity of RSV to readily penetrate through AM to establish and propagate the infection along the conducting airways.

We next assessed the mobility of RSV in the same AM treated with Mota‐MT to a final concentration of just 1 µg mL^−1^. Across nearly all AM samples, regardless of the mobility of RSV in the native AM, the motion of nearly all RSV virions became strongly hindered, with the vast majority moving less than their diameter (*d* ≈0.01 µm, Figure 1A) over the course of the 20 sec videos (Movie S2, Supporting Information). This is reflected by both a marked reduction in the <Deff> by ≈fivefold and fast‐moving populations by ≈20–30‐fold upon the addition of Mota‐MT (Figure 1A,B). The muco‐trapping effect was dose‐dependent; in the same AM specimens, increased concentration of Mota‐MT in AM samples led to smaller fraction of fast‐moving RSV (Figure 1C). We further tested palivizumab that was engineered with high levels of G0F to be mucotrapping (“Pali‐MT”), and found it similarly hindered the mobility of RSV in AM (Figure S1, Supporting Information). These results were in good agreement with our publications on muco‐trapping antibodies for SARS‐CoV‐2^[^ ^26^ ^]^ and Ebola.^[^ ^25^ ^]^

To further investigate the role of Fc N‐glycans in mAb‐mediated trapping of RSV in AM, we prepared Mota possessing different levels of G0F glycosylation. We found that addition of Mota possessing ≥ 30% G0F into AM was highly effective at trapping RSV in AM specimens that otherwise saw large fractions of mobile RSV (Figure 1D,E), across both AM collected from adults and from pediatric populations. Indeed, averaged across both adult and pediatric mucus samples, whereas the addition of mAb with 0% G0F resulted in comparable <Deff> to control AM (0.107 ± 0.104 µm^2^ s^−1^ versus 0.074 ± 0.073 µm^2^ s^−1^, respectively, *p* = 0.19), the addition of Mota‐MT with 30% and 60% G0F resulted in significant reductions of <Deff> to 0.019 ± 0.016 µm^2^ s^−1^ and 0.012 ± 0.012 µm^2^ s^−1^, respectively (*p* < 0.01). Likewise, the fraction of fast‐moving RSV in AM samples treated with Mota with 0% G0F (11.3±10.0%) was not statistically significantly different compared to in Control AM (9.1±8.8%, see Movie S3, Supporting Information) (*p* = 0.28), whereas the addition of Mota‐MT with 30% and 60% G0F reduced the fraction of fast‐moving RSV to 3.3 ± 3.8% (*p*<0.05) and 0.4 ± 0.4%, respectively (*p* < 0.01). These results substantiate the role of mAb Fc‐N‐glycosylation in mediating effective “muco‐trapping” in human AM.

### 2.2 Muco‐Trapping mAb Quickly Clears RSV from the Mouse Lung

**Figure 2:**
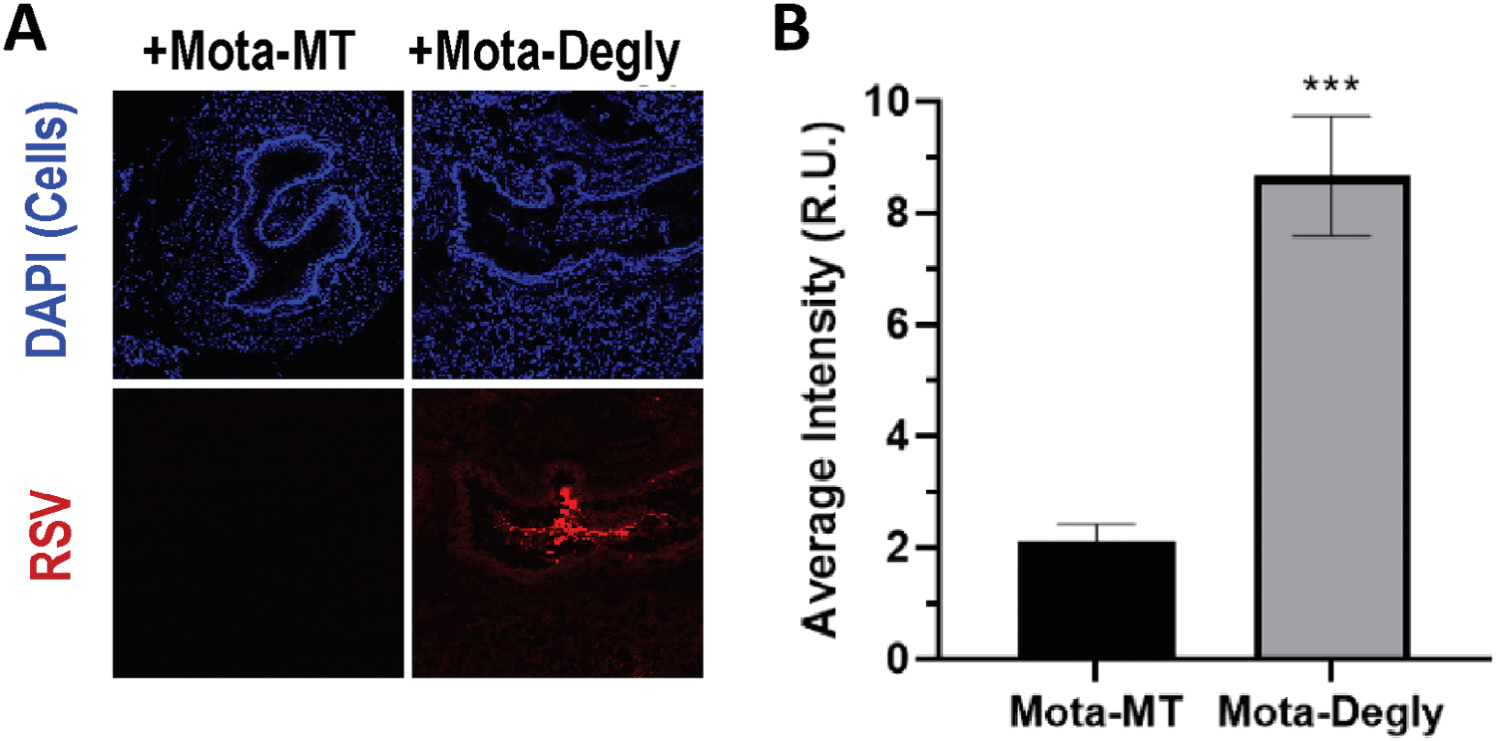
Mota‐MT mediated clearance of RSV from mouse airways depends on Fc‐glycosylation. Mice were first dosed with fluorescent RSV (red) intranasally, followed by intranasal instillation of Mota‐MT or Mota‐Degly 30 mins later, and then finally sacrificed 30 min later (*n* = 2 animals per treatment condition, from which were harvested sections from ≈8 spatially distinct regions of each lung). The lungs were immediately collected and cryo‐preserved to facilitate cryo‐sectioning. A) Representative images capturing the presence of fluorescent RSV in mice treated with either Mota‐MT (≥60% G0F) or Mota‐Degly (0% G0F). B) ImageJ quantification of the signal intensity from cryosection images similar to those shown in panel A, plotted as averages with 95% confidence intervals. *** represents *p*<.001.

Given the robust ability of Mota‐MT to trap RSV in AM *ex vivo*, we next sought to investigate whether the Fc‐mucin interactions with muco‐trapping mAbs would accelerate the clearance of RSV from the respiratory tract in mice. We first administered fluorescently‐tagged RSV‐A2 using a PennCentury microsprayer, followed by either Mota‐MT or deglycosylated version of the same mAb (i.e., Mota‐Degly) 30 min later, also administered using microsprayer. Mota‐Degly is able to bind RSV F protein with the same affinity and can likewise engage Fc receptors, and simply lacks the glycans needed to interact with mucins. Mice were sacrificed 30 mins after the mAb was dosed, and the trachea and airways were harvested and rapidly frozen for cryo‐sectioning. In this mouse model of airway clearance, topical delivery of Mota‐MT led to nearly complete elimination of the virus (**Figure** **2**A,B). In contrast, under otherwise identical settings, mice treated with Mota‐Degly were significantly less able to facilitate clearance of RSV from the airways, with ≈fourfold more RSV fluorescence than mice treated with Mota‐MT (Figure 2B). We saw similar results with Pali‐MT, which led to a ≈sixfold reduction in the amount of RSV fluorescence present in the airways compared to mice receiving PBS controls (Figure S2, Supporting Information), reaching levels comparable to mice that were pretreated with Pali‐MT 30 mins prior to RSV dosing. These results confirmed that muco‐trapping mAbs can facilitate rapid elimination of RSV from the airways.

### 2.3 Intranasal Administration of Muco‐Trapping mAb Reduces Viral Titers in Cotton Rats

**Figure 3:**
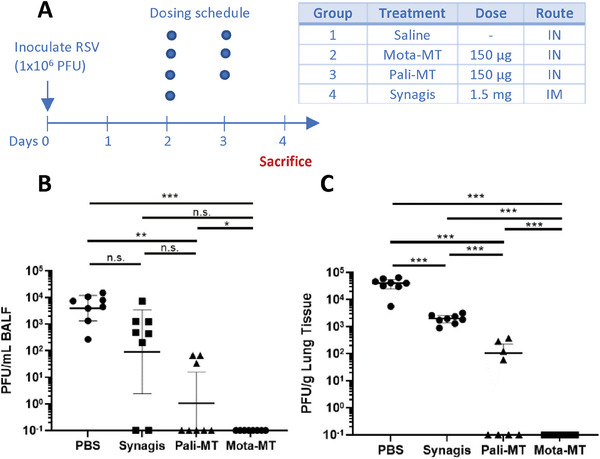
Intranasal Pali‐MT and Mota‐MT effectively suppressed RSV viral load in cotton rats. A) Study design. Cotton rats were infected with RSV Strain A2 (10^6^ PFU per rat) on Day 0. Starting on Day 2, the infected animals with treated daily with either intranasal saline, intranasal Pali‐MT, intranasal Mota‐MT, or intramuscular palivizumab (Synagis) given once. Two days later (i.e., Day 4), animals were sacrificed for assessment of viral load in (B) bronchoalveolar lavage fluid (BALF) and C) lung tissues by plaque assay. (*n* = 8 per treatment group in all panels). *, **, and *** represent *p*<.05, <.01, and <.001 on one‐sided *t* tests that were not adjusted for multiple comparisons.

We next assessed the efficacy of muco‐trapping mAbs against RSV infections in cotton rats, a markedly more permissive model for RSV transmission than mice.^[^ ^34^ ^]^ We first inoculated cotton rats with RSV Strain A2 (100 µL; 10^6^ PFU per cotton rat administered intranasally) on Day 0, followed by daily intranasal dosing of Mota‐MT or Pali‐MT, or one‐time intramuscular dosing of palivizumab (Synagis), starting at 48 h post infection. We harvested the lungs as well as collected bronchoalveolar lavages (BALF) on Day 4 to quantify viral load.^[^ ^35^ ^]^ In good agreement with our hypothesis, treatment with intranasal Mota‐MT at a dose of just 0.33 mg kg^−1^ led to complete reduction of infectious viral titers in the BALF (**Figure** **3**B) and in lung tissues (Figure 3C) within just 2 days of treatment. There was also a substantial reduction in the viral titers of animals receiving Pali‐MT. In contrast, treatment with intramuscular Synagis achieved only ≈1‐log reduction in viral titer relative to saline control, or roughly 1000‐fold less efficacy compared to Mota‐MT.

### 2.4 Muco‐Trapping mAbs can be Stably Nebulized Using a Vibrating Mesh Nebulizer

While intranasal delivery (using a dropper to instill liquid into the nares) is a common technique for topical administration in small rodents, achieving uniform delivery to both the upper and lower respiratory tracts is likely essential to maximize therapeutic benefits in humans, particularly since infections can likely initiate in both the nasal turbinate as well as the conducting airways in the lung. We thus sought to determine whether we could stably nebulize Mota‐MT while generating aerodynamic particle size distributions (APSD) suitable for delivery throughout the respiratory tract (i.e., 2–5 µm). As a proof‐of‐concept, we nebulized Mota‐MT using an Aerogen Solo vibrating mesh nebulizer and collected the generated aerosols into a Copley Next Generation Impactor (NGI). Analysis of inertial impaction results, based on distribution of antibody mass with respect to impaction stage cutoff diameter, showed a Mass Median Aerodynamic Diameter (MMAD) of 4.4 ± 0.2 µm (GSD = 2.5 ± 0.1), with a fine particle fraction (FPF) of 56.2% ± 1.1% (indicative of the percentage of mAb nebulized into aerosol particles with diameter smaller than 5.39 µm) (Figure S5, Supporting Information). The MMAD of 4.4 µm matches well with the performance predicted by Aerogen, and falls within the typical range of particle sizes that are efficiently deposited into the upper and lower airways.^[^ ^36^ ^]^ To illustrate nebulization of Mota‐MT is device‐agnostic, we also evaluated a second nebulizer, the InnoSpire Go, which has received 510(k) registration for human use. We found comparable APSD profile, with MMAD of 4.75 ± 0.11 µm, GSD of 2.20 ± 0.06 µm, FPF of 53.3 ± 0.86% (Figure S6, Supporting Information). These results underscore our ability to generate aerosols containing our muco‐trapping mAbs in the desirable size range for pulmonary delivery.

To test the physical stability and binding potency of Mota‐MT after nebulization, we characterized Mota‐MT pre‐ and post‐nebulization with the Aerogen Solo nebulizer, testing by ELISA (to measure binding affinity to RSV F protein) and by HPLC (to determine percent main peak by molecular weight). On ELISA, pre‐ and post‐nebulized Mota‐MT had EC50 of 11.3 ± 1.0 ng mL^−1^ and 11.2 ± 0. 4 ng mL^−1^, respectively. On HPLC, percentages in the main peak (by mass) for pre‐ and post‐nebulized Mota‐MT were 98.5% ± 0.1% and 98.4% ± 0.4%, respectively (*p* = 0.13). Together, these findings confirm that, after nebulization, the binding affinity and stability of Mota‐MT were fully preserved.

### 2.5 Nebulized Mota‐MT Effectively Treats RSV Infections in Neonatal Lambs

Finally, we sought to evaluate the efficacy of nebulized Mota‐MT against established RSV infections in neonatal lambs, the most representative preclinical model of RSV infection.^[^ ^37^, ^38^, ^39^ ^]^ Although animals such as cotton rats are susceptible to RSV infection and provide a convenient model of infection, their pulmonary architecture and disease physiology are markedly different than RSV‐infected infants. In contrast, neonatal lambs have been shown to be a faithful model of pediatric RSV infections due to similarities in developmental (e.g., extent of alveolar development), structural (e.g., lung size and airway branching), physiological and immunologic features.^[^ ^39^, ^40^ ^]^ Indeed, neonatal lambs are susceptible to infection by human RSV, and the RSV‐infected animals present many clinical symptoms and signs of pathology that are similar to those seen in RSV‐infected infants.^[^ ^39^, ^41^, ^42^ ^]^

**Figure 4:**
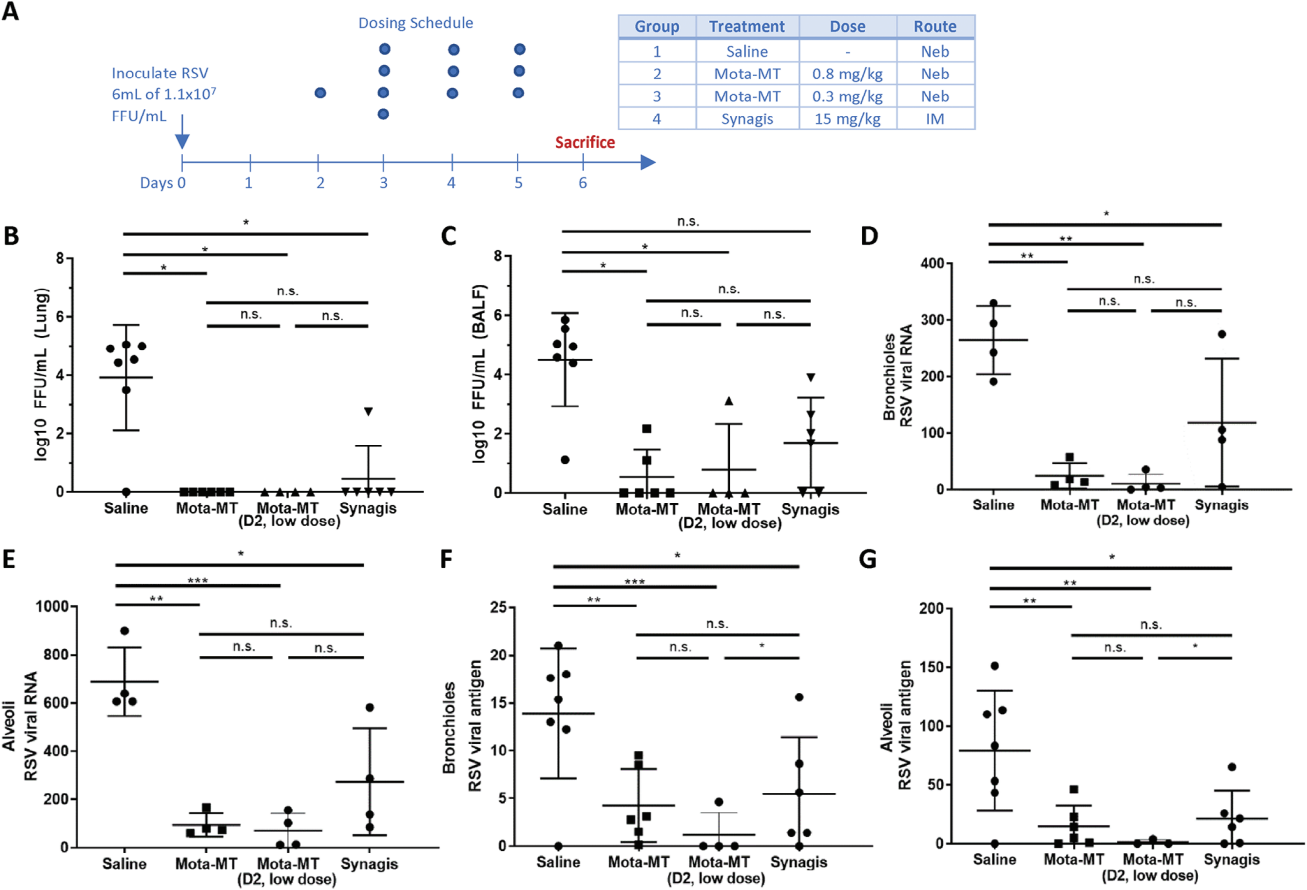
Treatment with nebulized motavizumab provides rapid protection against RSV infection. A) Study design: neonatal lambs (3–5 days old) were infected with RSV (Memphis R37 strain) on Day 0, and initiated treatment on 3 post‐infection (except for one group, which was initiated on D2, marked in panels). Groups treated with motavizumab received daily nebulized treatment on Days 3–5 or 2–5. Group “Synagis IM” received a single IM dose on Day 3. B) Fluorescent focus‐forming units (FFU) per gram of lung homogenates. C) FFU per mL BALF. D) viral RNA in bronchioles, as observed by RNAscope detection of RSV mRNA in FFPE tissue sections in situ. E) viral RNA in alveoli, by RNAscope. F) Viral antigen in bronchioles, by immunohistochemistry (IHC). G) Viral antigen in alveoli, by IHC). *, **, and *** represent *p*<.05, <.01, and <.001, respectively. Animal numbers per group varied across treatment conditions and analyses, but are represented as individual datapoints for each animal in each group for each analysis.

We first inoculated infectious RSV (Memphis R37 strain) into 3–5‐day‐old lambs on Day 0, and initiated treatment 3 days later when the infection was around peak viral titers in the lung; this treatment schedule is consistent with the timing of development of early symptoms and clinical diagnosis in infants. Infected lambs were treated with nebulized Mota‐MT, either dosed at ≈0.8 mg kg^−1^ starting on Day 3, or at ≈0.3 mg kg^−1^ starting on Day 2. We incorporated IM Synagis dosed on Day 3 as a positive control for systemic mAb, and nebulized saline as vehicle control. The negative control group consisted of uninfected lambs treated with nebulized saline. The animals were then sacrificed on Day 6 for a variety of virological and histological assessments. A summary of dosages and treatment schedules is shown in **Figure** **4**A.

We first assessed the viral titers in the lungs and BALF. In animals treated with vehicle control, we observed high levels of viral titers in both the lung tissues and BALF reaching nearly 10^5^, confirming extensive RSV infection (Figure 4B,C). Impressively, we observed a >10 000‐fold reduction in viral titers with no detectable infectious virus in the lung tissues of any of the animals treated with nebulized Mota‐MT, regardless of the day that treatment was initiated. We also saw highly effective reduction in the viral titers in the BALF obtained from the same lambs, with ≈3 orders of magnitude lower viral titers relative to saline control on average. There were either no detectable virus or titers below the assay's detection threshold in 5 of 6 animals in the Mota‐MT Day 3 group, and in 3 of 4 animals Mota‐MT Day 2 group. The difference in viral titers in the BALF or lung tissues in animals that received nebulized Mota‐MT versus IM Synagis did not reach statistical significance. Due to the low numbers of animals per group inherent with large animal studies, we were limited in our ability to detect smaller differences in efficacy across treatment groups.

The reduction in viral titers is consistent with RSV viral RNA levels in the alveolar and bronchial tissues: animals that were treated with nebulized Mota‐MT had markedly lower RSV RNA than vehicle control and Synagis IM in the bronchioles (Figure 4D) and in the alveoli (Figure 4E). To confirm these findings, tissue section immunohistochemically stained with anti‐RSV to determine the amount of RSV antigen present in the tissues and in situ localization. Animals that received nebulized Mota‐MT had significantly lower levels of RSV antigen in their tissues relative to control animals in both the alveoli (Figure 4F, *p*<0.01) and bronchioles (Figure 4G, *p* < 0.01). Together, these findings suggest that treatment of RSV‐infected lambs with nebulized Mota‐MT affords highly effective suppression of viral burden within just 3 days of treatment compared to controls, even when the treatment was delayed until around the time of peak viral titer, at 3 days after RSV infection was established in the lower respiratory tract. In the context of a human infection, lower respiratory tract disease would represent a relatively late stage of infection.

**Figure 5:**
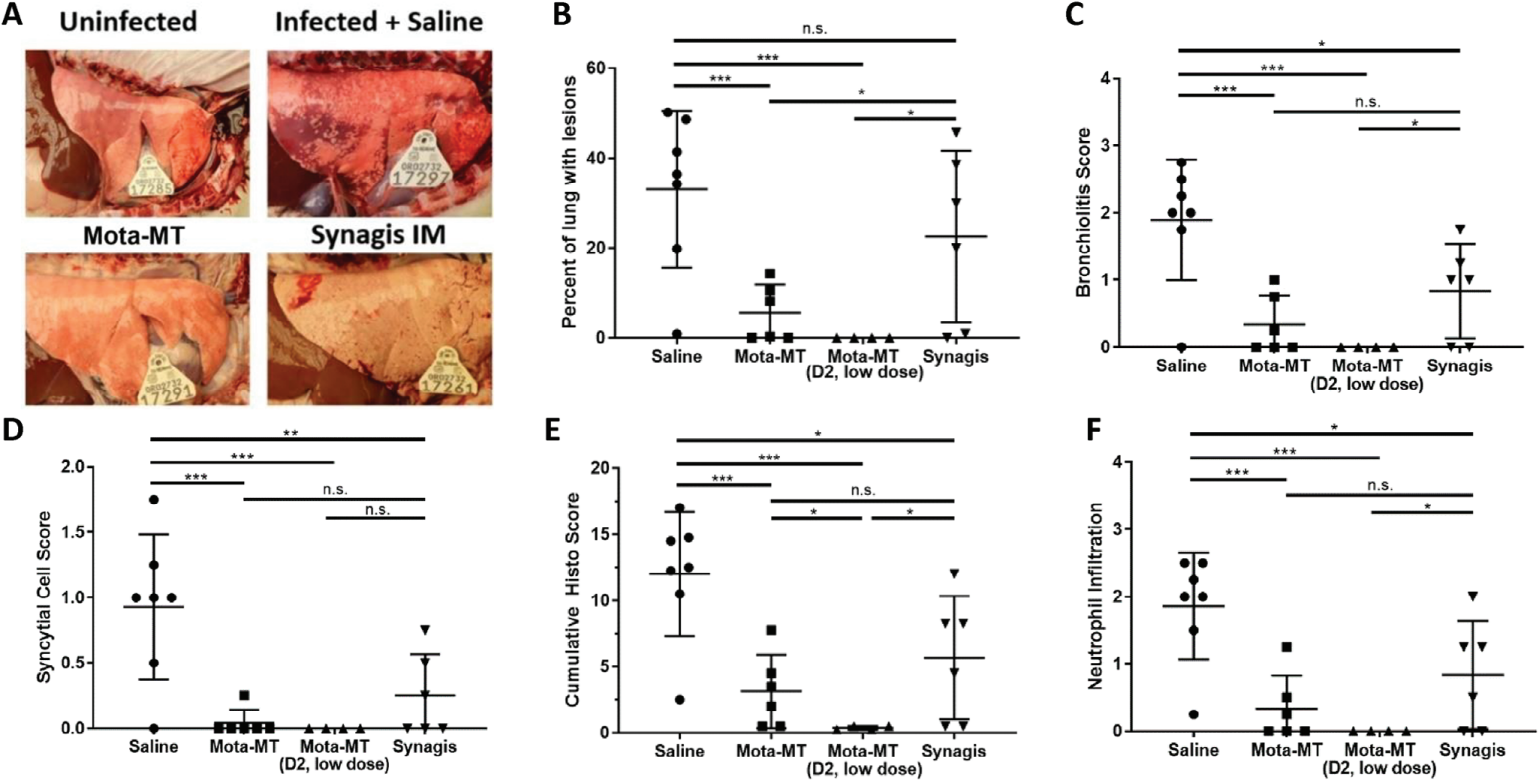
Nebulized Mota‐MT provides potent protection against histopathologic evidence of disease. A) Gross images of lamb lungs. B) Gross lung lesion scores, quantified by percent of lung tissue involvement. C) Bronchiolitis scores, D) Histopathologic scoring of syncytial cells, E) Lung consolidation scores, determined on a scale with a non‐linear, 0–4 scoring system, as previously discussed^[^ ^79^ ^]^ F) Neutrophil infiltration of lung tissues. *, **, and *** represent *p*<0.05, 0.01, and 0.001, respectively. N.S. denotes no significant difference in score. Histopathological scoring systems were adapted directly from Table 2 of,^[^ ^37^ ^]^ and increasing progression from score 0, to 1, to 2, does not represent a directly linear increase in severity. However, group averages and statistical comparisons were calculated on the basis of these scores, and groups with higher scores across these metrics exhibited worse signs and symptoms of disease. Animal numbers per group varied across treatment conditions and analyses, but are represented as individual datapoints for each animal in each group for each analysis.

Also assessed were a variety of RSV pathology, including gross and microscopic lung lesions, all analyzed in a blinded fashion. Gross lesion scores reflect the percent of lung parenchymal involvement of gross RSV lesions and can be considered a general marker of pulmonary inflammation and damage. As expected, RSV‐infected animals treated with saline had high gross lesion scores, reflective of extensive infection‐induced pulmonary damage. Animals treated with IM Synagis also had high gross lesion scores, no different than vehicle control, consistent with its lack of efficacy in clinical trials when used to treat established RSV infections. In sharp contrast, treatment with nebulized Mota‐MT was highly effective at reducing gross lesions, particularly when treatment was initiated early on Day 2 (**Figure** **5**B). Many of the Mota‐MT‐treated animals had no visible RSV‐induced lesions (score = 0).

Beyond gross lesion scores, lung microscopic pathology were assessed using a scoring system adapted from^[^ ^43^ ^]^ that provides a near‐logarithmic assessment ranging from 0–4: for instance, a bronchiolitis score of 1 represented minimal lesions in a few bronchioles per 20× field, a score of 2 represented lesions in <10% of the airway lumen, and a score of 3 represented lesions in 10–50% of the airway.^[^ ^43^ ^]^ Thus, the scores should not be interpreted as a linear reflection of pathology. We found that animals in the treatment groups had significant improvements in bronchiolitis (Figure 5C), syncytial cell score (Figure 5D), and neutrophil infiltration (Figure 5F), which led to an overall significant reduction in the accumulative histo lesion score, which represents the sum of all microscopic changes in the lung section (Figure 5E). Impressively, many of the markers of lung pathology in animals treated with Mota‐MT were indistinguishable from uninfected control lambs. These results underscore nebulized Mota‐MT as an effective treatment against RSV infections.

## 3 Discussion

Given longstanding challenges of vaccine hesitancy and breakthrough infections, treatments that can be quickly administered following emergence of symptoms, and that can effectively prevent RSV‐induced bronchiolitis and hospitalizations, are sorely needed. Since RSV viral load in the respiratory tract is predictive of disease severity in RSV‐infected infants, therapeutic modalities that can directly reduce the viral load and virus‐associated antigens in the airways should translate to reduced disease severity.^[^ ^44^ ^]^ Here, by directly delivering mAbs via inhalation that can trap RSV viruses directly into the respiratory tract, we demonstrate we can quickly reduce RSV viral titers by 4–5 orders of magnitude, and, in the process, abrogate associated inflammatory damage to levels nearly indistinguishable to uninfected animals within just 3 days of treatment. Compared against the timeline of human infections, delaying treatment in the lamb study until 3‐days after infection of the lower respiratory tract with RSV, around the time of peak viral load, represents a challenging model for treatment, and the observed reductions in viral load and pathology suggest a wide window of time for the successful initiation of therapy. The efficacy observed compares favorably to JNJ‐53718678 evaluated in the same neonatal lamb model, which achieved ≈2‐log reduction in viral load versus vehicle control^[^ ^45^ ^]^ in the same neonatal lamb model and was subsequently advanced into Phase 2 clinical studies. Since symptoms from RSV infections usually present for 3–5 days before reaching peak severity,^[^ ^46^ ^]^ providing multiple opportunities for medical intervention,^[^ ^47^ ^]^ the inhaled muco‐trapping mAb approach may offer not only faster symptom resolution but also reduced rates of RSV‐hospitalization in humans.

One of two distinct features of our approach, namely harnessing Fc‐mucin bonds in mAb engineering, has been underappreciated and thus underexplored. Indeed, few studies have investigated the role of IgGs in reinforcing mucus as the first line of defense, particularly since early work showed that IgGs possessed what was thought to be negligible interactions with mucins. By investigating how IgG impacts the mobility of viruses in mucus, we discovered that the array of virion‐bound IgG can generate strong binding *avidity* to mucins that virtually immobilizes virion‐IgG complexes in mucus. This muco‐trapping mAb function is particularly suited to quickly intervene in RSV infections, as shedding progeny virus into the AM and the diffusion of virus across mucus to infect the next cell are key steps in the spread of the infection.^[^ ^14^, ^48^, ^49^ ^]^ By crosslinking RSV to mucins, we are likely able to halt the spread of the infection as soon as mAbs are inhaled and deposited into the respiratory tract. While the lamb study described in this work was not adequately powered to reveal the contributions by different mAb effector functions, the potential importance of muco‐trapping Fc may be partially inferred by comparing against earlier work on inhaled delivery of camelid‐derived “nanobodies” in the same RSV‐challenge lamb model.^[^ ^50^ ^]^ While they observed effective neutralization of the virions, we found animals treated with “muco‐trapping” mAb also afforded lower levels of pulmonary inflammation, as reflected by gross lesions and consolidation scores.

The second distinct feature of our approach is delivery by inhalation, which contrasts sharply to the longstanding focus on systemic delivery (IV, SC, IM). Surprisingly, despite the fact that RSV and other respiratory virus concentrates in the airways,^[^ ^15^, ^16^, ^51^ ^]^ inhaled delivery of human or humanized mAbs against RSV had not been investigated in large animal studies or advanced into the clinic.^[^ ^52^ ^]^ The lack of interest in inhaled dosing may be a consequence of decades‐old work that found mAbs were at high risk of being denatured by jet nebulizers, loss of activity, aggregation, and thus perceived risk of immunogenicity. Other forms of inhaled delivery of therapeutic proteins, including dry powder inhaler formulations, require extensive formulation development to avoid hydroscopic growth and agglomeration, even after being successfully de‐aggregated from micronized materials.^[^ ^53^, ^54^ ^]^ Nevertheless, these challenges have now been overcome by advances in vibrating mesh nebulizer technology and greater familiarity in formulation development. We have now been able to formulate a variety of mAbs for stable nebulization, meeting specifications required by regulatory agencies to advance into clinical trials.^[^ ^55^ ^]^ Nebulization can deliver a large dose of mAb to the airways in minutes^[^ ^26^, ^55^ ^]^ while keeping the total volume of treatment solution relatively low^[^ ^56^ ^]^ and achieving uniform dispersion throughout the upper and lower respiratory tract.^[^ ^56^, ^57^, ^58^, ^59^ ^]^ This makes nebulization particularly well suited for delivery of antiviral mAbs, as it is not possible to know a priori where the virions are distributed within the respiratory tract when treatment is initiated.

The consequence of delivering mAbs systemically is that they distribute both slowly and inefficiently into the respiratory tract; this may partially account for the lack of efficacy observed in clinical trials evaluating the use of Synagis or motavizumab as treatment for RSV infections.^[^ ^33^, ^60^ ^]^ Indeed, studies in non‐human primates showed as little as 1/500th of the systemically administered mAb dose actually distributes into the lung.^[^ ^61^ ^]^ In a human influenza‐challenge study, patients were treated with 50 mg kg^−1^ of CR6261, an anti‐influenza mAb.^[^ ^62^ ^]^ Cmax in nasal swab samples was achieved only on Day 2 or 3 after dosing and reached a level of only ≈0.6 µg mL^−1^ CR6261, a concentration that is below its IC_50_. In contrast, mAbs administered directly to the respiratory tract achieve local Cmax immediately after dosing, which is often orders of magnitude greater than the IC_50_ of the mAb, even when dosed at lower total dose compared to systemic treatment. For instance, the nasal Cmax achieved with nebulizing a 90 mg mAb dose^[^ ^55^ ^]^ was over 1500‐fold higher than Cmax achieved with dosing 3000 mg+ of CR6261.^[^ ^55^ ^]^ This allows mAbs dosed by inhalation to intervene much earlier in the viral replication cycle, before infection spreads further into the deep lung and before a vicious feed‐forward inflammatory cycle is initiated.^[^ ^63^ ^]^

In the cotton rat studies, we saw that intranasal dosing with Pali‐MT resulted in two distinct phenotypes: most animals were fully protected, but a handful only saw partial reduction of viral titers. In contrast, intranasal dosing with Mota‐MT fully protected all animals. We believe this is likely attributed to the inherent variable pulmonary distributions with intranasal dosing in rodents, a notorious problem in the field. Specifically, we believe the partial protection observed occurred as a result of inadequate antibody dosed to the local portion of the lung with RSV infections in those animals. Similar variability in pulmonary distribution likely also occurred with Mota‐MT, but was mitigated by the 20‐40‐fold greater activity of the molecule. This motivated our decision to advance Mota‐MT into the neonatal lamb studies. We are unable to discern the relative importance of inhaled delivery versus muco‐trapping at this time, since mAbs that do not facilitate muco‐trapping can still confer protection by multiple mechanisms, including neutralization and ADCC, and thus very large group size would likely be necessary to reveal additional contribution by muco‐trapping. Nonetheless, we consider the comparison largely academic. Since G0/G0F N‐glycosylation patterns do not abolish other well‐established Fc effector functions, and elevated levels of G0/G0F can be readily achieved from bioproduction using Chinese Hamster Ovary cells, there is little reason to not exploit the muco‐trapping effector function in advancing mAbs for treating acute respiratory infections while retaining other Fc effector functions. Likewise, given the far superior PK and dosing convenience with inhaled versus systemic delivery, there is little reason to not pursue inhaled delivery of mAbs for treating respiratory infections. Beyond improved PK/distribution, inhaled delivery offers several other key advantages. First, inhaled dosing can take place anywhere, including in the comfort of one's own home. This convenience should enable greater ease of access by patients and facilitate earlier treatment. Second, by avoiding infusion, we bypass the need for infusion chairs, infusion centers or the need for qualified healthcare professionals to administer the dose and monitor the patient. This substantially reduces the burden on the healthcare system, as lack of infusion facilities was a major limiting factor to the adoption of systemic mAb‐based treatments for patients with mild to moderate COVID.^[^ ^49^ ^]^ The lower burden on the healthcare system, as well as the much smaller dose of mAb needed for effective treatment, should also translate to lowering the overall costs of treatment.

The translational potential of inhaled muco‐trapping mAbs is supported by our recent Phase 1 clinical trial evaluating the safety, tolerability, and pharmacokinetics of IN‐006, an inhaled muco‐trapping mAb against SARS‐CoV‐2.^[^ ^55^ ^]^ There were no serious adverse events in the study, and all enrolled participants completed the study without treatment interruption or discontinuation, underscoring the overall highly favorable safety profiles of inhaled mAbs. We believe this safety is an important advantage, as a large number of small molecule antivirals that were previously advanced into clinical development for RSV had stalled due to safety concerns. Patients in the highest dose group achieved mean nasal fluid concentrations in excess of 900 µg mL^−1^ 30 min after dosing, and retained >5 µg mL^−1^ 22–24 h later, supporting a once‐per‐day inhaled dosing regimen where highly inhibitory mAb concentrations are maintained through trough. More impressively, the gradual rise in serum Cmax over several days implies high mAb levels were retained in the deep lung for many days after a single inhaled dose, likely at concentrations that were 3+ orders of magnitude greater than the typical IC_50_ of potent antiviral mAbs. These data, combined with the impressive efficacy observed in our lamb study, suggests inhaled muco‐trapping mAb may likely offer significant benefit against RSV disease in humans.

Building off the COVID experience, we believe the field is heading toward an at‐home treatment following at‐home diagnosis paradigm. Rapid antigen tests that can simultaneously diagnose multiple different infections are now increasing available. Following a positive diagnosis, a treatment can be quickly prescribed to facilitate early intervention to prevent pneumonia and other pulmonary complications. The key to such a model is a treatment that is exceptionally safe with no concerns for drug‐drug interactions and can be easily administered, including to infants and older adults suffering from dysphagia. Such a paradigm not only minimizes the burden on the healthcare system, but also reduces the need to bring infected patients into healthcare facilities. We believe that inhaled delivery of muco‐trapping mAbs can meet this need. We further envision the approach to be highly applicable to any respiratory viruses where the predominant route of entry and shedding is through the apical membrane, a list that spans a diverse array of viruses including influenza,^[^ ^64^, ^65^ ^]^ SARS‐CoV‐1 and SARS‐CoV‐2,^[^ ^66^, ^67^ ^]^ human metapneumovirus,^[^ ^68^ ^]^ seasonal coronavirus such as HKU1 and NL63,^[^ ^69^, ^70^ ^]^ and parainfluenza virus.^[^ ^71^, ^72^ ^]^

## 4 Experimental Section

### Human Airway Mucus Samples

Human AM was obtained from healthy adult or pediatric patients intubated for general anesthesia during elective surgery (for a non‐pulmonary indication), following a protocol that was deemed non‐human subjects research by the UNC‐CH IRB (Not research, no protocol number). After surgery, the endotracheal tube was removed from the patient, and mucus coating the tube was collected by gentle centrifugation.^[^ ^73^ ^]^ AM that was non‐uniform in color or consistency or that had visible blood contamination was discarded. Samples were treated with protease inhibitor immediately after collection to minimize potential enzymatic degradation, and stored at 4°C prior to use in microscopy studies to assess the ability of mAbs to reduce the diffusion of fluorescently labeled RSV particles in the fresh AM (typically conducted within 8 h of sample storage at 4°C). Measurement of the diffusion of the fluorescent virions was carried out as previously described.^[^ ^23^, ^24^, ^25^, ^74^ ^]^

### Animal Study Ethical Approvals

All animal studies described in this work were conducted under the guidance of IACUC regulations and in line with pre‐approved protocols to ensure minimal use of animals and to avoid discomfort. The cotton rat studies were carried out by IBT Biosciences. The neonatal lamb studies were carried out by LambCure and were performed in accordance with the animal welfare standards of the Legacy Research Institute which are in accordance with the Association for Assessment and Accreditation of Laboratory Animal Care (AAALAC) regulations (AAALAC #000992). The study also obtained approval from the Institutional Biosafety Committee (IBC) at the Legacy Research Institute under protocol (119‐2018).

### High Resolution Multiple Particle Tracking for Quantifying RSV Mobility in Human AM

Purified RSV was fluorescently labeled as previously described.^[^ ^19^ ^]^ Different mAbs and RSV were added to AM in a custom‐made small‐volume glass chamber, gently mixed, and sealed.^[^ ^75^ ^]^ The samples were then incubated for ≈15 min at 37°C prior to microscopy. Particle motion was recorded using an EMCCD camera (Evolve 512; Photometrics, Tuscon, AZ) mounted on an inverted microscope (AxioObeserver D1; Zeiss, Thornwood, NY) equipped with an Alpha Plan‐Apo 100x/1.46 NA objective, environmental (temperature and CO2) control chamber and an LED light source (Lumencor Light Engine DAPI/GFP/543/623/690). Videos (512 ×512, 16‐bit image depth) were captured with MetaMorph imaging software (Molecular Devices, Sunnyvale, CA) at a temporal resolution of 66.7 ms and spatial resolution of 10 nm (nominal pixel resolution 0.156 µm per pixel). A minimum of 5 videos capturing a combined minimum of 100 particles on a frame‐by‐frame basis were captured for each condition/specimen. Particle trajectories were then obtained using a recently developed convolutional neural network.^[^ ^74^ ^]^ Time scale‐averaged MSDs and effective diffusivity were calculated by transforming particle centroid coordinates into timescale dependent MSDs with the formula <Δr^2^(τ)> = [x(t + τ) – x(t)]^2^ + [y(t + τ) – y(t)]^2^, where τ = time scale or time lag.

### Mouse Studies

For the studies of mAb‐mediated clearance in mice, we used female BALB/c mice, ≈6–10 weeks old (University of North Carolina at Chapel Hill, IACUC 18–034). Mice were anesthetized using isoflurane prior to intranasal instillation of fluorescent RSV at 25 µL using the PennCentury microsprayer, allowed to recover for 30 min, and then provided with intranasal instillation of mAb or control saline (25 µL via microsprayer). Mice were euthanized 30 min after the administration of treatment and the trachea and lungs were dissected. The trachea and lungs remained attached through dissection and were washed with PBS. The trachea and lungs were embedded in 100% optimal cutting temperature compound (OTC) to be frozen at −80°C. After overnight freezing, tissue samples were cryosectioned into 10 µm thick sections at −20°C and stained with DAPI. Images were captured with a fluorescent confocal microscope at the following excitation/emission spectrum: 10x, TRITC (559/603 nm) and DAPI (405/422 nm).

### Cotton Rat Studies

Mota‐MT was assessed in a cotton rat model of RSV infection. This model was established and validated using the RSV strain A2. Animals were infected intranasally on day 0. One group received topical Mota‐MT treatment at 48 and 72 h post‐infection and another group received an intramuscular injection of Synagis at 48 h post‐infection. Bronchoalveolar lavage fluid (BALF) and lung samples were analyzed for plaque‐forming units (PFUs) to quantify viral titers.

### Neonatal Lamb Model

PolyPay, Dorsett, or other cross lambs of both sexes in between 1 to 3 days of age (3‐6 kg per animal) were obtained in the State of Oregon, USA. Two to 14 animals per pen were housed under controlled conditions that included a 12 h day^−1^ artificial light cycle (6 am – 6 pm). Animals were colostrum‐deprived and fed, ad‐libitum, iodide‐free lamb milk replacer (Milk Products Inc., Chilton, WI, USA) from birth until sacrifice. Water was provided in the reconstituted milk. Lambs were given Naxcel (Ceftiofur sodium, Pfizer) subcutaneously once‐daily to reduce/prevent secondary bacterial infections.

RSV‐infected lambs were kept in a separate room from uninfected animals, with separate ventilation units as well as separate entrances and exits. In all contexts, control lambs were manipulated first, followed by RSV lambs, to avoid infection.

### Production of Challenge Virus for Neonatal Lambs

RSV virus used in this study was Memphis 37 (M37) RSV, which is a wild type RSV‐A^[^ ^76^ ^]^ used previously in human clinical studies.^[^ ^77^, ^78^ ^]^ RSV were propagated in HEp‐2 cells and stored in −80°C in sucrose‐stabilizing media

### Viral Infection and mAb Treatment of Neonatal Lambs

Aerogen Solo nebulizers were used to administer virus (6 mL of RSV M37 strain at 1.1 × 10^7^ FFU mL^−1^ in media containing 20% sucrose) or control media (media from HEp‐2 cells lacking RSV containing 20% sucrose) to each lamb.

Motavizumab was shipped at 4°C to the lamb study facilities. Target dose calculations assumed ≈11% effective inhaled dose,^[^ ^79^ ^]^ i.e., for each 1 mL of liquid that was nebulized, 0.11 mL was assumed to be inhaled by the lambs, due to various inefficiencies such as accumulation of aerosol on the tubing between nebulizer and mask, the neonatal breathing pattern, as well as imperfect seals between the masks and lambs. mAbs and control solutions were administered using vibrating mesh nebulizers.

### Sample Collection from Lambs

From neonatal lambs, blood was collected from the external jugular vein with a syringe and dispensed into K2EDTA tubes, then kept cold before centrifugation within 2 h of collection (1600 g for 10 min at 4C). Plasma was stored at −80C until further analysis. The lungs were collected following sacrifice, photographed, and scored for gross lesion and samples were taken in 10% neutral buffered formalin for histopathological sectionss. BALF for qRT‐PCR assessment of viral load was collected immediately by rinsing the right caudal lobe with 5 mL of cold double‐modified Iscove's media (DMIM) containing 42.5% Iscove's modified Dulbecco's medium, 7.5% glycerol, 1% heat‐inactivated FBS, 49% DMEM, and 5 µg mL^−1^ kanamycin sulfate. The BALF was flushed through the major bronchus five times (with the single 5 mL aliquot) prior to final collection of ≈2–6 mL of fluid. BALF for assessment of mAb and urea concentrations was collected from the right middle lung lobe using five 1 mL flushes of 0.9% sterile normal saline, resulting in a total collection of ≈2–3 mL BALF.

### Infectious FFU Assay on BALF and Lung Tissue

Prior to use in the infectious focus assay, collected BALF samples was first microfuged at 1780 x g for 5 min to pellet any large debris, after which 800–850 µL of each clarified supernatant was removed and filtered at 0.45 µm using a Costar SPIN‐X filter (Fisher Scientific, Hanover Park, IL, USA). At this point, the BALF samples were considered ready for infectious focus assay.

In addition, prior to use in the infectious focus assay, 0.5 g of collected lung samples (pooled from 0.1 g from each of 5 lobes for each animal; right cranial, accessory, left cranial, left middle, and left caudal) were homogenized in 5 mL of DMIM on ice for 80 s using an OMNI TH homogenizer. One milliliter of each homogenate was microfuged at 1780 x g for 5 min to pellet large debris. Before filtration at 0.45 µm. At this point, the lung sample homogenates were considered ready for infectious focus assay.

Briefly, HEp‐2 cells were grown to 70% confluence in 12‐well culture plates in DMEM media supplemented to 10% heat‐inactivated fetal bovine serum (FBS) and 50 µg mL^−1^ kanamycin sulfate. Each filter‐clarified BALF sample was analyzed at full‐strength and at four additional serial‐dilutions of 1:10, 1:100, 1:1000 and 1:10000. Plates were incubated for 80 min in a CO2 incubator at 37°C and 5% CO2 with manual rocking every 20 min. 1 mL of culture medium was added to each well and cells were allowed to incubate for 48 h after which medium was removed and cells were fixed with 60% acetone/40% methanol solution for 1 min. The fixing solution was removed and plates were allowed to air‐dry for 2 min after which each well was rehydrated with 1 mL TBS‐0.05% Tween 20, pH 7.4‐7.6 (TBST) for 1 min with mild rotation. To block non‐specific binding, 1 mL of 3% BSA (Fisher Scientific, Hanover Park, IL, USA) in TBST was added to all wells at room temperature with gentle rocking for 30 min. Primary polyclonal goat anti‐RSV (all antigens) antibody (EMD Millipore, Billerica, MA) was diluted 1:800 in TBST containing 3% BSA; 325 µL of this was added to each well and plates were allowed to incubate overnight at 4°C with gentle rocking. The next day, plates were washed gently three times for 5 min each with TBST, then 325 µL secondary antibody [Alexa Fluor 488 F (ab’) 2 fragment of rabbit anti‐goat IgG (H+L), Molecular Probes/Life Technologies] diluted 1:800 in TBST containing 3% BSA was added to each well and allowed to incubate at room temperature for 30 min with gentle orbital rotation. Plates were rinsed two times for 5 min each with TBST and 1 mL of TBST was added back to each well prior to microscopic inspection. Plates were examined for the presence of fluorescing infectious foci using the FITC/GFP filter on an inverted stage fluorescence microscope (Olympus CKX41, Center Valley, PA, USA). Clusters of 5 or more fluorescing cells were counted as single infectious focal events.

### Immunohistochemistry for RSV Antigen in Formalin‐Fixed Paraffin‐Embedded Lung Tissue Sections

Immunohistochemistry for visualization of RSV antigen was performed on 5 µm‐thick formalin‐fixed paraffin‐embedded (FFPE) lamb lung tissue sections taken from the right and left cranial, left middle, and left caudal lung lobes of each animal in accordance with methods published previously.^[^ ^79^ ^]^ Briefly, slides were heated for 15 min at 58°C, deparaffinized in xylene, rehydrated through a series of graded ethanol (100%, 95%, 70%) solutions including an intervening step (between the 95% and 70% ethanol steps) to block for endogenous tissue peroxidase activity with 3% H_2_O_2_ in methanol for 15 min, finally ending up in double‐distilled H2O (ddH2O). Slides were then subjected to heated buffer antigen retrieval using TRIS‐EDTA pH 9.0 containing 0.05% Tween 20 in a pressure‐cooking device (Decloaking Chamber Plus, Biocare Medical, Concord, CA, USA) that heated the slides in retrieval buffer to 125°C within 18 min, then cooled them to 80°C within 22 min. Slides (still in retrieval buffer) were then cooled on ice for 20 min, rinsed with TBS‐0.05% Tween 20, pH 7.4 (TBST) and incubated in TBST for 5 min. The upper and lower portions of each slide were then wax‐penned (ImmEdge Pen, Vector Laboratories, Inc., Burlingame, CA, USA) to corral subsequent reagents. Using an OptiMax i6000 automatic staining machine (BioGenex, San Ramon, CA, USA) sections were blocked for 15 min with 3% bovine serum albumin (BSA) in TBST followed by blocking with 20% normal swine serum (NSS) (Life Technologies, Carlsbad, CA, USA) in TBST for another 15 minutes. Primary polyclonal goat anti‐RSV (all antigens) antibody were applied for 90 minutes at room temperature diluted 1:500 in TBST containing 10% NSS and 3% BSA. After rinsing with TBST, biotinylated rabbit anti‐goat secondary antibody (Kirkegaard‐Perry Labs, Gaithersburg, MD, USA) diluted 1:300 in TBST containing 10% NSS and 3% BSA will be applied for 45 min, after which slides will be rinsed with TBST, treated with 3% H2O2 in TBST for 25 min, rinsed with TBST, and then incubated with streptavidin‐conjugated HRP (Life technologies) diluted 1:200 in TBST for 30 min. After manual rinsing of the slides with TBST, color was manually developed in custom 12‐slide plastic containers (Antibody Amplifier containers, ProHisto, LLC, Columbia, SC, USA) by applying Nova Red (Vector Laboratories, Inc.) for ≈90 s followed by copious rinses with ddH2O, counterstaining with Harris’ hematoxylin (for 2 min), bluing with alkaline Scott's water (for 1 min), dehydration (back through graded ethanol solutions, ending with xylene), and cover slipping with Permount mounting medium (Sigma, St. Louis, MO, USA). Twenty unique 10× fields on each slide (containing two sections each) will be assessed for RSV antigen staining by counting positively‐stained cells within bronchioles and alveoli. The number of cells staining for RSV per field will be then assigned a score according to the scale: 0 = no positive cells, 1 = 1–10 positive cells, 2 = 11–39 positive cells, 3 = 40–99 positive cells, 4 = >100 positive cells. The Kruskal‐Wallis (non‐parametric) statistical test will be used to assess the results for significance.

### Urea Measurement to Estimate Extent of Lung Fluid Dilution

During collection of BALF, buffer was added to the lungs to rinse out lung fluid, which was subsequently used to estimate the concentration of mAb achieved in the airways. During this process, the lung fluid is inherently diluted by rinsing buffer. To correct for that dilution factor, the concentration of urea was measured in the BALF and in the terminal plasma; urea concentrations in lung fluid and plasma should be equivalent at steady state, and therefore dividing the plasma concentration of urea by the BALF concentration of urea provides an estimate of the extent of dilution of lung fluid. Urea concentrations were measured using a colorimetric assay kit per manufacturer's instructions (Abcam ab83362).

### Next Generation Impactor Studies to Assess Aerosol Particle Size of Nebulized mAb Formulations

Generally, USP <1601> was adhered to for generation of data and calculation of mass median aerodynamic diameter (MMAD) and geometric standard deviation (GSD) after nebulization of mAb. Briefly, the Next Generation Impactor (NGI; MSP Corp, MN, USA) and collection stages were precooled to 4°C for at least 90 min prior to experiments. The Aerogen Solo vibrating mesh nebulizer was loaded with enough mAb solution to ensure replicates could be performed while avoiding sputtering (i.e., remaining above the manufacturer's minimum recommended volume). A custom mouthpiece, molded to the nebulizer/NGI inlet interface, was used to affix the nebulizer to the inlet with a tight seal. A solenoid in line with the NGI and vacuum (set to 15 L min^−1^) was used to collect sufficient nebulized mAb at a given concentration. The nebulizer was actuated, and the solenoid was switched on to begin collection. Following nebulization, the vacuum and nebulizer were switched off and the NGI stages and inlet were removed. Quickly, the next set of stages and inlet were swapped in to perform a second replicate nebulization. Stages were washed with 5 mL of buffer and assayed by UV‐visible spectroscopy at A280 for mAb mass deposition, which was used for calculation of MMAD, GSD, and FPF.

### Statistical Analysis

Data were generally not transformed, normalized, or evaluated for outliers prior to analysis. Unless otherwise indicated in the figure legend, data are presented as arithmetic mean ± SD in graphical formats, and the sample sizes for respective comparisons are noted in the figure legends. Unless otherwise indicated, statistical comparisons consisted of 2‐sided Student's *t*‐test, assuming unequal variance (heteroscedastic). For all comparisons, the alpha was set as 0.05, and p values less than 0.05 were considered to represent significant differences. Calculations were not adjusted for multiple comparisons in the context of multiple treatment groups. Microsoft Excel was used for statistical analysis.

## Conflict of Interest

S.K.L. is founder of Mucommune, LLC and currently serves as its interim CEO. S.K.L is also founder of Inhalon Biopharma, Inc, and currently serves as its CSO, Board of Director, and Scientific Advisory Board. S.K.L. has equity interests in both Mucommune and Inhalon Biopharma; S.K.L.‘s relationships with Mucommune and Inhalon are subject to certain restrictions under University policy. The terms of these arrangements are managed by UNC‐CH in accordance with its conflict of interest policies. M.D.M., E.F., B.N., B.F., T.M., S.A, and M.A. have equity interests in Inhalon Biopharma.

## Supporting information

Supporting Information

Supplemental Movie 1

Supplemental Movie 2

Supplemental Movie 3

## Data Availability

The data that support the findings of this study are available from the corresponding author upon reasonable request.
