# Supplementary material for: Inhaled “Muco‐Trapping” Monoclonal Antibody Effectively Treats Established Respiratory Syncytial Virus (RSV) Infections
Source: Adv Sci (Weinh). 2024 Jan 15;11(12):2306729. doi: 10.1002/advs.202306729 (PMC10966576; doi:10.1002/advs.202306729)
Supplement: Supplementary file 1 — Supporting Information [file ADVS-11-2306729-s002.pdf]

ADVANCED SCIENCE
Open Access

Supporting Information

for *Adv. Sci.*, DOI 10.1002/advs.202306729

Inhaled "Muco-Trapping" Monoclonal Antibody Effectively Treats Established Respiratory Syncytial Virus (RSV) Infections

*Morgan D. McSweeney, Sarhad Alnajjar, Alison M. Schaefer, Zach Richardson, Whitney Wolf, Ian Stewart, Pun Sriboonyapirat, Justin McCallen, Ellen Farmer, Bernadette Nzati, Sam Lord, Brian Farrer, Thomas R. Moench, Priya A. Kumar, Harendra Arora, Raymond J. Pickles, Anthony J. Hickey, Mark Ackermann and Samuel K. Lai**

Supplemental Figures

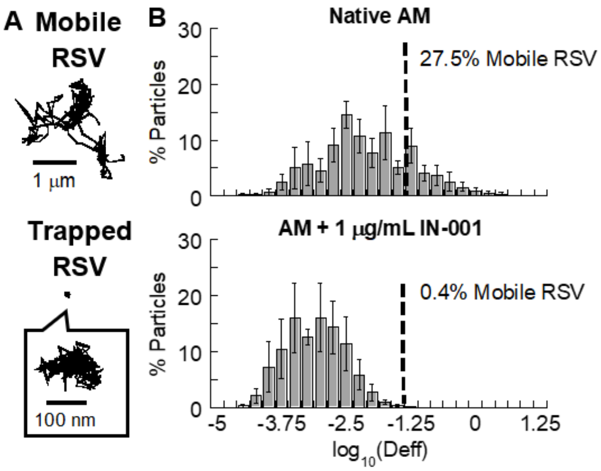

Supplemental Figure 1. Addition of Pali-MT to AM reduces mobility of fluorescent RSV virions in AM. (A) Representative traces of RSV in native AM or in AM treated with Pali-MT. **(B)** Distributions of the logarithms of individual particle effective diffusivities (D_{eff}). Decreased mobility of RSV by Pali-MT is reflected by a shift toward smaller D_{eff}.

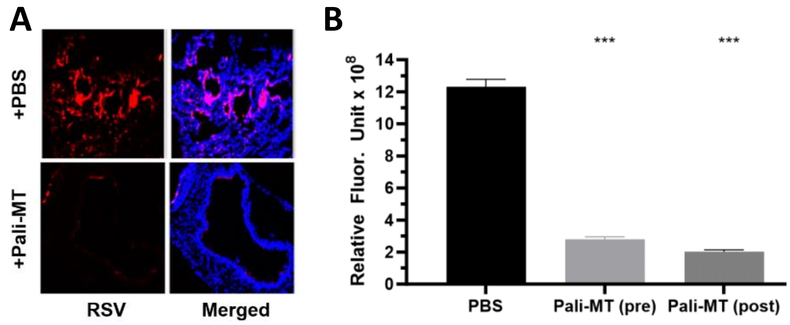

Supplemental Figure 2. **Pali-MT clears RSV from the airways in mice.** Mice were first dosed with fluorescent RSV (red) intranasally, followed by intranasal instillation of various mAbs 30 mins later, and then finally sacrificed 30 minutes later. The lungs were immediately collected and cryo-preserved to facilitate cryo-sectioning. **(A)** Representative images capturing the presence of fluorescent RSV in mice treated with either PBS (top row) or pali-MT (bottom row). Cells are stained with DAPI. **(B)** Quantitative analysis of the signal intensity from cryosection images by Image J, similar to those shown in (A). Pali-MT (Pre) denotes mice that dosed with Pali-MT 30 mins prior to RSV virions. Data are plotted as means with SEM.

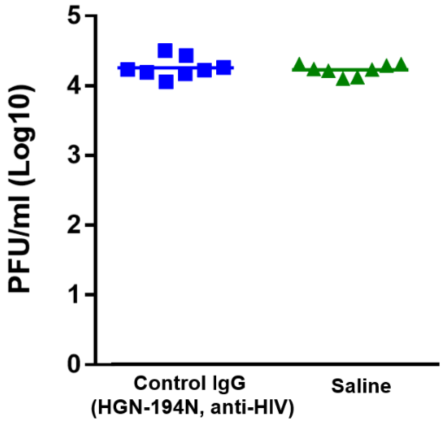

Supplemental Figure 3. Intranasal dosing of non-specific control IgG does not reduce RSV viral titers in lungs in cotton rats. To ensure that the antigen-specificity of Mota-MT was responsible for the observed reduction in viral titers, we compared treating RSV-infected cotton rats with either saline or HGN-194N, a neutralizing mAb against HIV. Both groups of animals exhibited similar viral load in their lungs on Day 4 post-infection, suggesting effective suppression of RSV titers by Mota-MT is not attributed to non-specific inhibition of RSV by human IgGs.

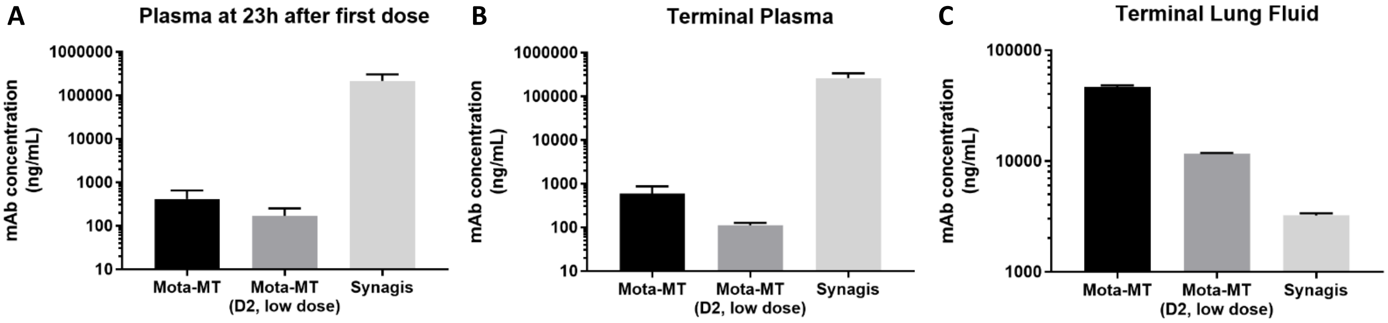

Supplemental Figure 4: Concentration of Mota-MT and Synagis in plasma and lung fluid of neonatal lambs determined by ELISA. Concentration of mAbs in plasma samples collected **(A)** 23 hours after the first dose of mAb, and **(B)** on Day 6 at the time of sacrifice, as well as in **(C)** Lung Fluid on Day 6 at the time of sacrifice. BALF was collected by rinsing the lungs with normal saline. The extent of dilution of lung fluid in BALF was determined by comparing the urea concentrations in terminal plasma and BALF, and the reported concentrations are corrected for that estimated BALF dilution factor. Error bars represent standard deviation.

A

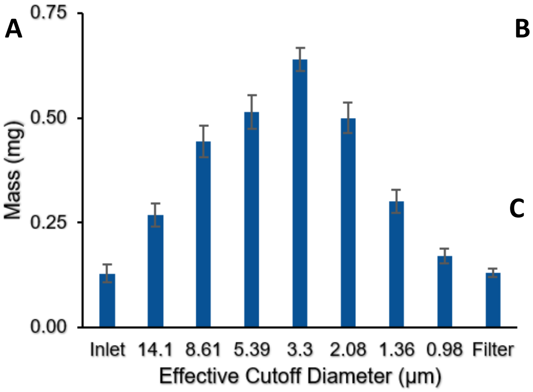

B

Mota-MT concentration	MMAD	GSD	FPF (<5.39 µm)
5.4 mg/mL	4.4 ± 0.2 µm	2.5 ± 0.1	56.2 ± 1.1%

C

ELISA EC_{50} (ng/mL)		HPLC (main peak, %)		HPLC (low MW, %)		HPLC (high MW, %)	
pre	post-neb	pre	post-neb	pre	post-neb	pre	post-neb
11.3 ± 1.0	11.2 ± 0.4	98.5 ± 0.1	98.4 ± 0.2	0.1 ± 0.0	0.1 ± 0.0	1.4 ± 0.1	1.6 ± 0.2

Supplemental Figure 5. Aerosol particle size distribution of Mota-MT after nebulization via Aerogen Solo nebulizer. Mota-MT at a formulation concentration of 5.4 mg/mL was nebulized via Aerogen Solo (mesh size 10) in three separate experiments to measure APSD using a Copley NGI. **A)** Mass of mAb deposited on each stage of the NGI over the course of a 1.5 minute actuation are shown to demonstrate the relative distribution of particle sizes, suitable for delivery to both the upper and lower respiratory tract. **B)** Summary statistics for APSD data collected over n=3 studies). **C)** Stability of Mota-MT pre- and post-nebulization with the Aerogen Solo nebulizer, as measured on RSV F protein coated ELISA for EC_{50}, as well as on size exclusion HPLC for main peak, low molecular weight (MW), and high MW peaks by mass.

A B

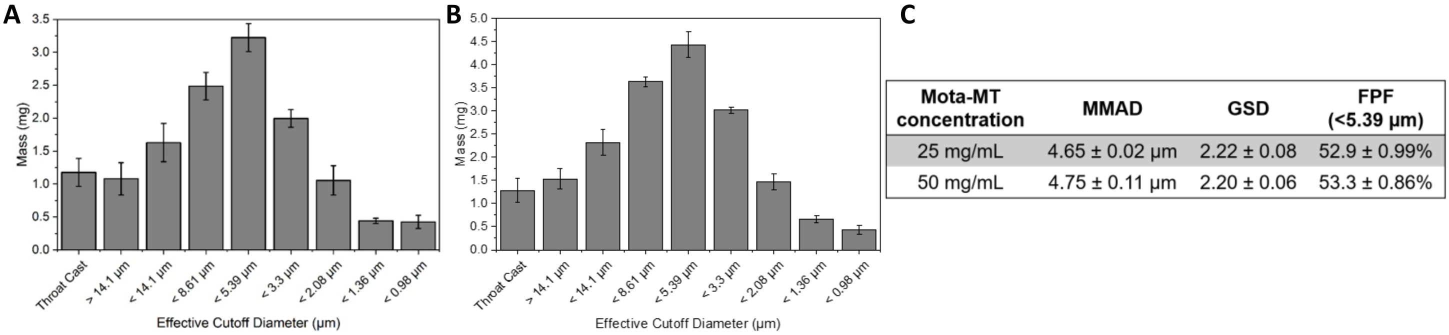

C

Mota-MT concentration	MMAD	GSD	FPF (<5.39 µm)
25 mg/mL	4.65 ± 0.02 µm	2.22 ± 0.08	52.9 ± 0.99%
50 mg/mL	4.75 ± 0.11 µm	2.20 ± 0.06	53.3 ± 0.86%

Supplemental Figure 6. Aerosol particle size distribution of Mota-MT after nebulization via InnoSpire Go nebulizer. Mota-MT at a formulation concentration of **A)** 25 mg/mL and **B)** 50 mg/mL was nebulized via InnoSpire Go in three separate experiments to measure APSD using a Copley NGI. Mass of mAb deposited on each stage of the NGI over the course of a 1 minute actuation are shown. **C)** Summary APSD statistics at each concentration.
